# Development and Validation of Prognostic Model in Transitional Bladder Cancer Based on Inflammatory Response-Associated Genes

**DOI:** 10.3389/fonc.2021.740985

**Published:** 2021-10-07

**Authors:** Zhiwen Xie, Jinming Cai, Wenlan Sun, Shan Hua, Xingjie Wang, Anguo Li, Juntao Jiang

**Affiliations:** ^1^ Department of Urology, Shanghai General Hospital, Shanghai Jiao Tong University School of Medicine, Shanghai, China; ^2^ Department of Geriatrics, Shanghai General Hospital, Shanghai Jiao Tong University, School of Medicine, Shanghai, China; ^3^ Department of Urology, The Fifth Peoples Hospital of Zunyi, Guizhou, China

**Keywords:** transitional bladder carcinoma, inflammatory response, prognostic model, risk score, drug sensitive, qRT-PCR

## Abstract

**Background:**

Bladder cancer is a common malignant type in the world, and over 90% are transitional cell carcinoma. While the impact of inflammatory response on cancer progression has been reported, the role of inflammatory response-associated genes (IRAGs) in transitional bladder cancer still needs to be understood.

**Methods:**

In this study, IRAGs were download from Molecular Signature Database (MSigDB). The transcriptional expression and matched clinicopathological data were separately obtained from public databases. The TCGA-BLCA cohort was used to identify the differentially expressed IRAGs, and prognostic IRAGs were filtrated by univariate survival analysis. The intersection between them was displayed by Venn diagram. Based on least absolute shrinkage and selection operator (LASSO) regression analysis method, the TCGA-BLCA cohort was used to construct a risk signature. Survival analysis was conducted to calculate the overall survival (OS) in TCGA and GSE13507 cohort between two groups. We then conducted univariate and multivariate survival analyses to identify independently significant indicators for prognosis. Relationships between the risk scores and age, grade, stage, immune cell infiltration, immune function, and drug sensitivity were demonstrated by correlation analysis. The expression level of prognostic genes *in vivo* and *in vitro* were determined by qRT-PCR assay.

**Results:**

Comparing with normal tissues, there were 49 differentially expressed IRAGs in cancer tissues, and 12 of them were markedly related to the prognosis in TCGA cohort for transitional bladder cancer patients. Based on LASSO regression analysis, a risk model consists of 10 IRAGs was established. Comparing with high-risk groups, survival analysis showed that patients in low-risk groups were more likely to have a better survival time in TCGA and GSE13507 cohorts. Besides, the accuracy of the model in predicting prognosis is acceptable, which is demonstrated by receiver operating characteristic curve (ROC) analysis. Age, stage, and risk scores variables were identified as the independently significant indicators for survival in transitional bladder cancer. Correlation analysis represented that the risk score was identified to be significantly related to the above variables except gender variable. Moreover, the expression level of prognostic genes *in vivo* and *in vitro* was markedly upregulated for transitional bladder cancer.

**Conclusions:**

A novel model based on the 10 IRAGs that can be used to predict survival time for transitional bladder cancer. In addition, this study may provide treatment strategies according to the drug sensitivity in the future.

## Introduction

Bladder cancer is a common malignant type in the world, and over 90% are transitional cell carcinoma, namely, bladder urothelial carcinoma (BUC), which accounts for the majority of primary bladder cancer (1). In addition to the type of non-muscle invasive tumor, muscle-invasive tumor is the other type of bladder cancer, which is categorized by the extent of tumor infiltration (2). Although 80% of bladder cancer are first diagnosed as non-muscle invasive tumor, up to 80% of them progress into muscle-invasive tumor after initial treatment within 5 years (2). However, metastasis in patients with muscle-invasive bladder cancer is easier to happen and has poor prognosis (3). Thus, it is imperative for us to determine practical biomarkers to predict BUC in patients at an early stage.

The connection between inflammation and cancer has been well established (4). Inflammation could not only inhibit tumors but also promote cancers (5). Recently, numerous studies indicated that inflammatory response regulates the development and progression of cancer, which has attracted increasing attention from researchers (6).

Since inflammatory biomarkers in the blood are easy to detect, researchers can explore their role in cancers (7). The indicators of inflammatory response, such as thrombocytosis, leukemoid, hypercalcemia, plasma fibrinogen, and D-dimer, as prognostic biomarkers for bladder cancer have been demonstrated (8, 9). In addition, the value of inflammatory indexes including lymphocyte-to-monocyte ratio, platelet-to-lymphocyte ratio, and neutrophil-to-lymphocyte ratio were estimated in new bladder cancer cases. Moreover, studies showed that these markers were the independent predictors for OS in bladder cancer (10–12). In the Glasgow prognosis model, C-reactive protein and albumin were independent prognostic factors in tumors (13). To develop a comprehensive prognostic model, increasing studies suggested that it would be a good choice for researchers to combine various acute phase proteins and inflammatory indexes. Furthermore, some IRAGs playing an important role in the metastasis of bladder cancer have been reported (14). However, the correlation between IRAGs and the prognosis of transitional bladder cancer still needs to be elucidated.

We first retrieved transcriptional expression and matched clinicopathological data of patients with transitional bladder cancer from TCGA and GEO databases in this study. Based on differentially expressed IRAGs, we then established and validated the risk model by TCGA and GSE13507 cohorts. Next, the possible mechanisms, which they were involved in, were explored by single sample gene set enrichment analysis (ssGSEA). Besides, associations between cancer stemness, tumor chemoresistance, immune infiltrate types, and the risk score were analyzed. Finally, qRT-PCR assay was performed to determine the levels of prognostic genes *in vivo* and *in vitro*.

## Material and Methods

### Data Collection and Preparation

Transcriptional expression profile and matched clinico-pathological data were obtained from The Cancer Genome Atlas (TCGA) database, which was made up of 409 transitional bladder cancers and 19 normal adjacent tissues, to establish the model. One hundred sixty-five transitional cell carcinoma samples were used to validate the model, which were download from GSE13507 cohort (http://www.ncbi.nlm.gov/geo/query/acc.cgi?acc=GSE13507). Apart from the above, 200 inflammatory response-associated genes were acquired from Molecular Signature Database (http://gsea-msigdb.org) (**Supplementary Table S1**).

### Identification of Differentially Expressed and Prognostic Inflammatory-Response-Associated Genes in TCGA Cohort

Limma R package was used to identify differentially expressed IRAGs in TCGA cohort, which were defined as those with a false discovery rate <0.05 and a |fold change| > 2. After eliminating the patient data with no survival time, the IRAGs with prognosis in TCGA cohort was determined by univariate Cox analysis, and Benjamini–Hochberg (BH) correction method was used to adjust the p-value.

### Establishment and Validation of a Prognostic Model

To avoid overfitting and to construct a novel gene signature in the study, LASSO regression analysis was used, the algorithm of which was conducted with glmnet R package to select and shrink variables for excluding the variables with a regression coefficient equal to 0. Then, an interpretable model was established according to the non-zero regression coefficients in TCGA cohort, and the optimum λ was selected in 10-fold cross-validation. We calculated the risk scores for each patient by summarizing the product of the expression level of each IRAGs and its corresponding regression coefficient and split BUC patients into two groups on the basis of risk scores in TCGA and GSE13507 cohort. We then conducted survival analysis to determine their different OS by using the R Survminer package and time‐dependent ROC analysis by using the survival and timeROC R package to assess prediction accuracy.

### Prognostic Implication of the Risk Score in TCGA Cohort

For tumor stage, age, gender, and risk scores variables, multivariate Cox analyses, after the initial screening by univariate cox analysis, were conducted to assess the implication of prognosis. Moreover, the relationship between risk scores and other variables were determined by correlation analysis.

### Immune Infiltration and Tumor Immune Microenvironment

We calculated the immune and stromal scores to evaluate the cell infiltration levels in transitional bladder cancer. The relationships between risk score and those cells were analyzed by Spearman correlation analysis. To assess the difference in immune infiltration subtype in the two groups, two-way ANOVA analysis was performed. In addition, the feature of tumor stemness cell were downloaded from TCGA tumor samples. To analyze the links between the feature of tumor stemness and risk score, Spearman correlation analysis was conducted.

### ssGSEA of the Gene Signatures

To explore the related pathways of the gene signatures in the TCGA cohort, ssGSEA was conducted in high and low scores, and BH method was used to calculate the adjusted p-value with the GSVA R package.

### Drug-Sensitive Analysis of the Gene Signatures

After logging into the CellMiner project page (https://discover.nci.nih.gov/cellminer), the transcriptional expression of NCI-60 human cancer cell lines was downloaded. The association between prognostic genes and drug sensitivity was determined by Pearson correlation analysis.

### qRT-PCR

Total RNAs of transitional bladder cancer tissues, adjacent normal tissues, T24, 5637, and SVHUC-1 cells were extracted by TRIzol (Novabio, China). Moreover, we obtained total RNAs of T24 and 5637 cells after treatment by 1 μm Dasatinib (15). We purchased these cell lines from Shanghai Chinese Academy of Sciences cell bank (Shanghai, China). RNA (1 μg) and PrimeScript RT kit (Novabio, China) were used to synthesize complementary DNA (cDNA). According to the manufacturer’s protocol, the amplification of cDNA was conducted by using SYBR Green reagent and ddH_2_O and analyzed by an ABI 7500 Real-Time PCR system (Applied Biosystems). All experiments were performed for three independent measures. Their primer sequences are shown in **Supplementary Table S3**.

### Statistical Analysis

We used R version 4.0.4 to conduct statistical analyses, and statistical significance was assumed at two-sided p-value below 0.05.

## Results

### Identification of Differentially Expressed Prognostic IRAGs in the TCGA Cohort

**Figure 1:**
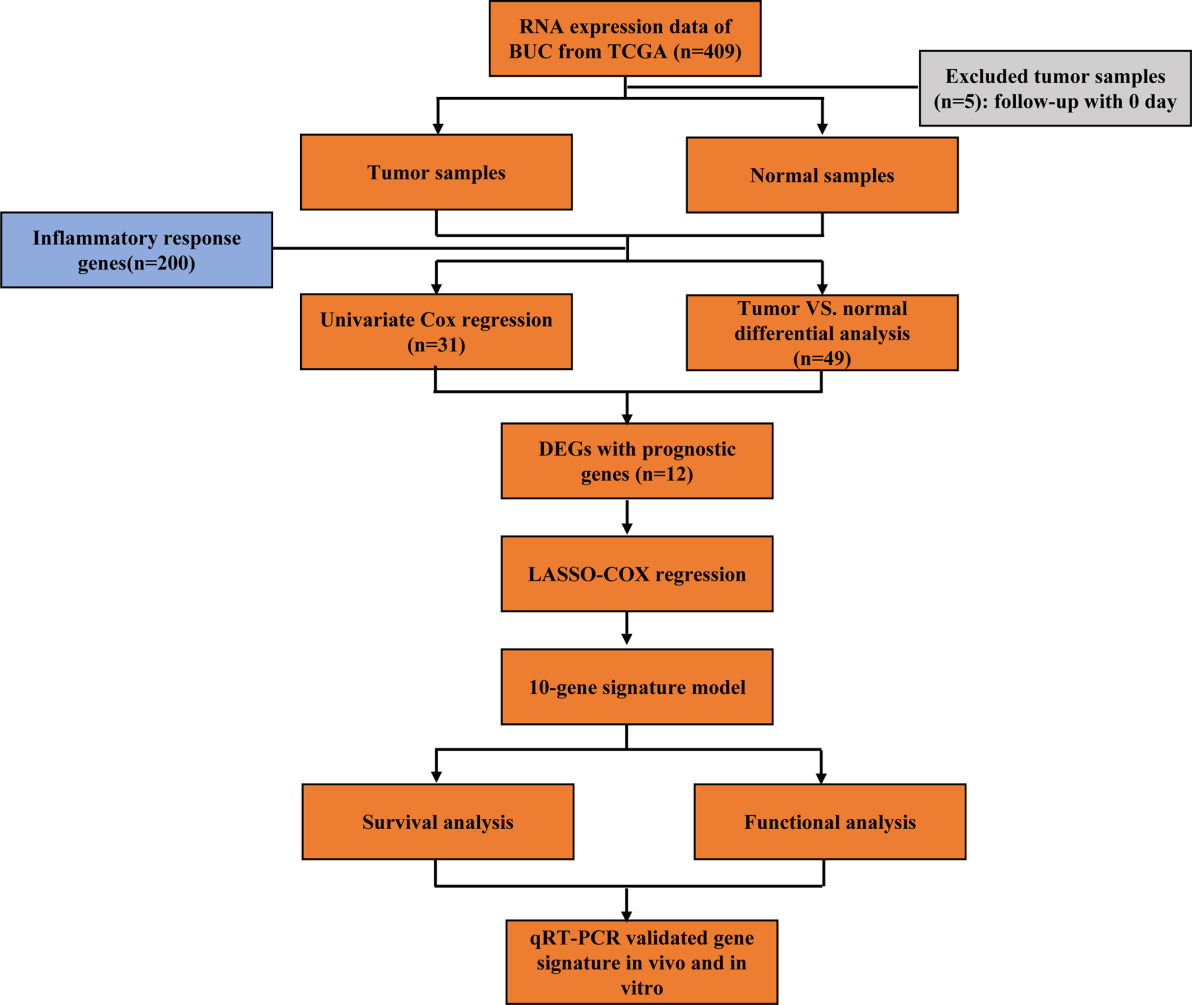
Flow chart of data collection, analysis, and experiment.

**Figure 2:**
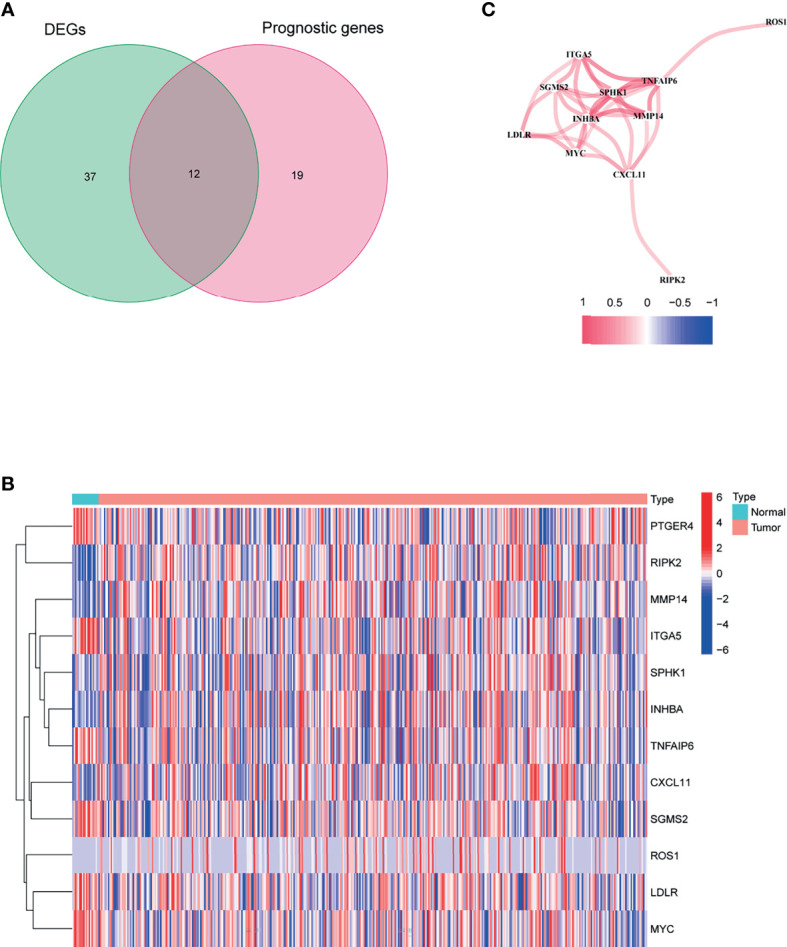
Identification of the candidate inflammatory response-associated genes in the TCGA cohort. **(A)** Venn diagram to identify DEGs between BLCA tissues and adjacent normal tissues. **(B)** The 13 overlapping genes expression between BLCA tissues and adjacent normal tissues. **(C)** The correlation network of candidate genes.

**Figure 1** shows the flow chart of this study. There are 404 BLCA patients with survival statistics in the TCGA cohort. In addition, 200 IRAGs were downloaded from the Molecular Signature database. There were 49 IRAGs differentially expressed in tumor and normal tissues (**Supplementary Table S2**). Univariate regression analysis demonstrated that 31 IRAGs are correlated with OS (**Supplementary Figure S1**). Then, Venn diagram was used to screen the prognosis-related differentially expressed IRAGs (**Figure 2A**). Heatmap revealed their expression status in tumor and adjacent tissues (**Figure 2B**). **Figure 2C** represents the interactions of these signatures.

### Development of a Prognostic Model

As shown in **Figure 2C**, there are 12 candidate genes that were associated with prognosis.

**Figure 3:**
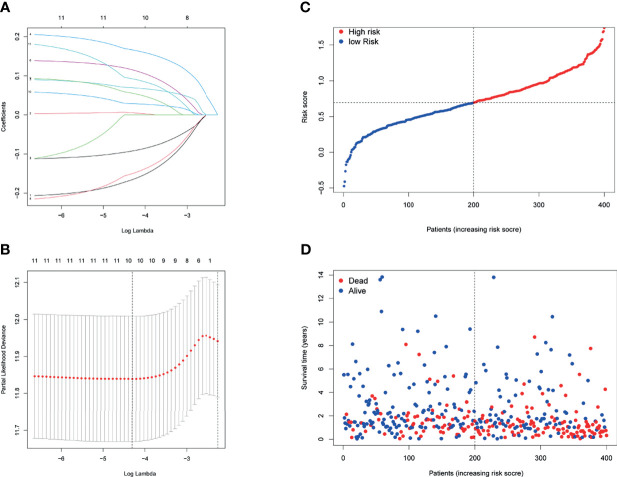
Prognostic analysis of the 10-gene signature model in the TCGA cohort. **(A)** LASSO coefficient expression profiles of 13 candidate genes. **(B)** The penalty parameter (λ) in the LASSO model was selected through 10 cross-validation. **(C)** The median value and distribution of the risk scores. **(D)** The distribution of OS status.

Then, a novel model consisting of 10 prognostic genes was developed through LASSO–Cox regression analysis (**Figures 3A, B**). The formula, risk score = (−0.166 * CXCL11) + 0.006 * INHBA + 0.166 * LDLR + 0.069 * MMP14 + 0.115 * MYC + (−0.09 * PTGER4) + (−0.150 * RIPK2) + 0.055 * SGMS2 + 0.029 * SPHK1 + 0.092 * TNFAIP6, was used to compute the risk score of each patient (**Figure 3C**). We then divided patients in the TCGA cohort into two groups by using the median risk scores (**Figure 3C**). Besides, scatter chart presented that patient in high-risk groups may have a worse outcome (**Figure 3D**).

### Prognostic Implication of the Risk Score

**Figure 4:**
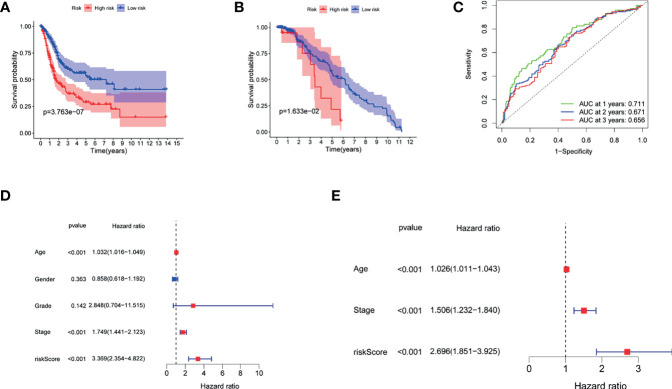
The role of the risk score in overall survival. **(A)** Kaplan–Meier curves for OS of patients in the high- and low-risk groups of TCGA. **(B)** Kaplan–Meier curves for OS of patients in the high- and low-risk groups of GEO13507 cohort. **(C)** AUC time-dependent ROC curves for OS in TCGA. **(D)** OS-related factors were screened by univariate cox regression analyses in TCGA. **(E)** OS-related factors were screened by multivariate cox regression analyses in TCGA.

**Figures 4A, B** demonstrate that patients with higher risk scores were more likely to suffer a markedly shorter OS than that of their counterpart in TCGA and GSE13507 cohorts, respectively. To investigate the predictive ability of the risk score for BUC patients, time-dependent ROC analysis was used, and the area under ROC (AUC) at 1, 2, and 3 years was 0.711, 0.670, and 0.655, respectively, in TCGA cohort (**Figure 4C**). In addition, the AUC at 1, 2, and 3 years was 0.669, 0.573, and 0.563, respectively, in GSE13507 cohort (**Supplementary Figure S2**). Moreover, our findings demonstrated that the risk score would be a significant variable for predicting prognosis by the univariate Cox regression analyses (HR = 3.369; CI = 2.354–4.822; p < 0.001) (**Figure 4D**). After eliminating the factors with p > 0.05, accompanying with stage and age, multivariate survival analyses showed that the risk score was negatively related to OS (HR = 2.696; CI = 1.851–3.925; p < 0.001) (**Figure 4E**).

### Clinical Implication of the Risk Score

**Figure 5:**
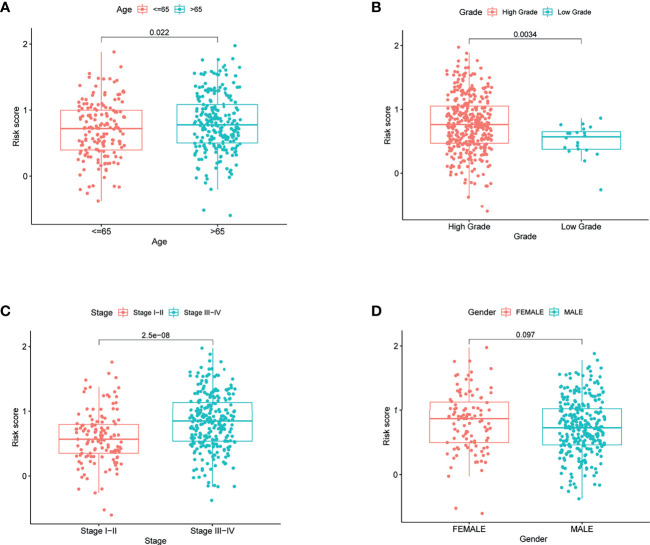
The risk score in different groups divided by clinical characteristics in TCGA. **(A)** Age, **(B)** grade, **(C)** stage, and **(D)** gender.

To elucidate the clinical implication of the risk score, clinical variables such as age, gender, grade, and stage variables were enrolled into the correlation analysis. Boxplot showed that patient with age >65, high grade, and stage III−IV are more likely have a high-risk score (**Figures 5A–C**). However, the relationship between gender variable and the risk score was not significant (**Figure 5D**).

### Immune Status and Tumor Microenvironment

**Figure 6:**
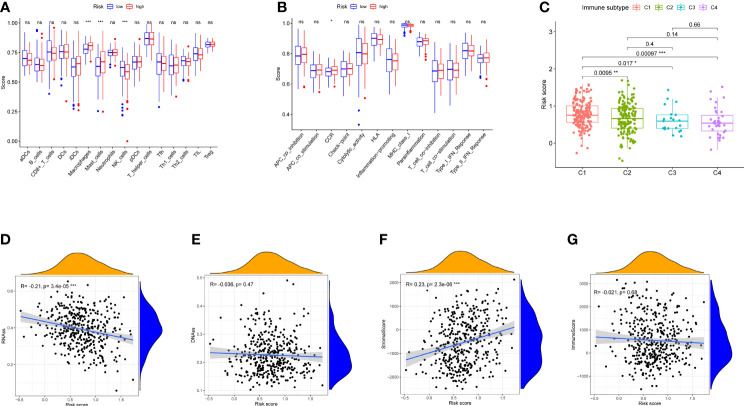
Immune status between different risk groups and the association between risk score and tumor microenvironment in TCGA cohort. **(A)** The scores of 16 immune cells are shown in boxplots. **(B)** Thirteen immune-related functions are shown in boxplots. **(C)** Comparison of the risk score in different immune infiltration subtypes. **(D–G)** The relationship between risk score and RNAss, DNAss, stromal score, and immune score. p-values are shown as ns, not significant, *p < 0.05, **p < 0.01, and ***p < 0.001.

Enrichment scores were calculated in different immune cell subpopulations of these two groups by ssGSEA. Compared with the low-risk group, we found that there are significant enrichment of mast cells, natural killer (NK) cells, and macrophages in the high-risk group (**Figure 6A**). Furthermore, the scores of immune functions between them were not significant except CCR (**Figure 6B**, p < 0.05).

Six types of immune infiltration types were added to determine the association with risk score, namely, wound healing (C1), interferon gamma (IFN-γ) dominant (C2), inflammatory (C3), lymphocyte depleted (C4), immunologically quiet (C5), and transforming growth factor beta (TGF-b) dominant (C6), were studied in our work, which may function as tumor promoting or repressing. There is no BLCA patient belonging to C5 and C6, which were not studied in our work. As shown in **Figure 6C**, infiltration of wound healing may happen in patients in the high-risk group, while the infiltrations of lymphocyte depleted, inflammatory, and IFN-γ dominant are more likely to appear in those with low-risk score (p < 0.05).

To measure the tumor stemness, RNA stemness score (RNAss) and DNA methylation pattern (DNAss) were calculated in this study. Correlation analysis represented that the risk score has negative correlation with RNAss (**Figure 6D**, p < 0.001), while there is no association with DNAss (**Figure 6E**, p > 0.05)

In addition, correlation analysis demonstrated that the risk score was positively associated with stromal score (p < 0.01), Nevertheless, no significant difference was observed in immune score (p > 0.05) (**Figures 6F, G**).

### Signaling Pathways in High- and Low-Risk Groups

**Figure 7:**
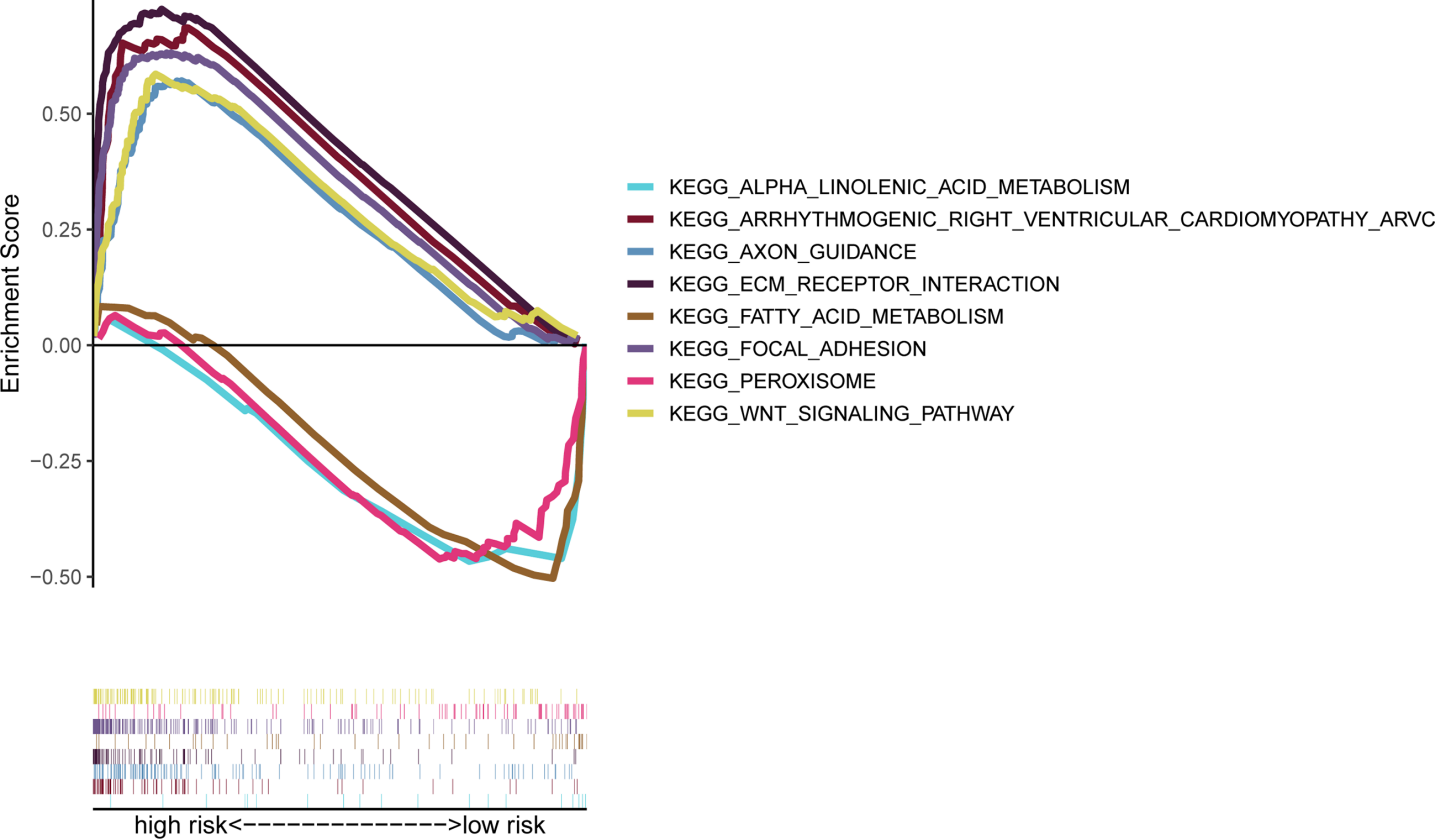
Gene set enrichment analysis of biological functions and pathways.

In TCGA cohorts, ssGSEA showed that signaling pathways, such as AXON GUIDANCE (normalized enrichment score NES = 2.20, p < 0.01), WNT_SIGNALING_PATHWAY [(NES) = 2.31, p < 0.01], ARRHYTHMOGENIC_RIGHT_VENTRICULAR_CARDIOMYOPATHY_ARVC (NES = 2.25, p < 0.01), FOCAL ADHESION (NES = 2.26, p < 0.01), and ECM_RECEPTOR_INTERACTION (NES = 2.22, p < 0.01), in the high-risk group were markedly enriched, while in the low-risk group, signaling pathways, such as FATTY_ACID_METABOLISM (NES = −1.71, p = 0.023), ALPHA_LINOLENIC_ACID_METABOLISM (NES = −1.61, p = 0.022), and PEROXISOME (NES = −1.62, p = 0.026) were significantly enriched (**Figure 7**).

### The Sensitivity of Prognostic Gene Expression to Chemotherapy

**Figure 8:**
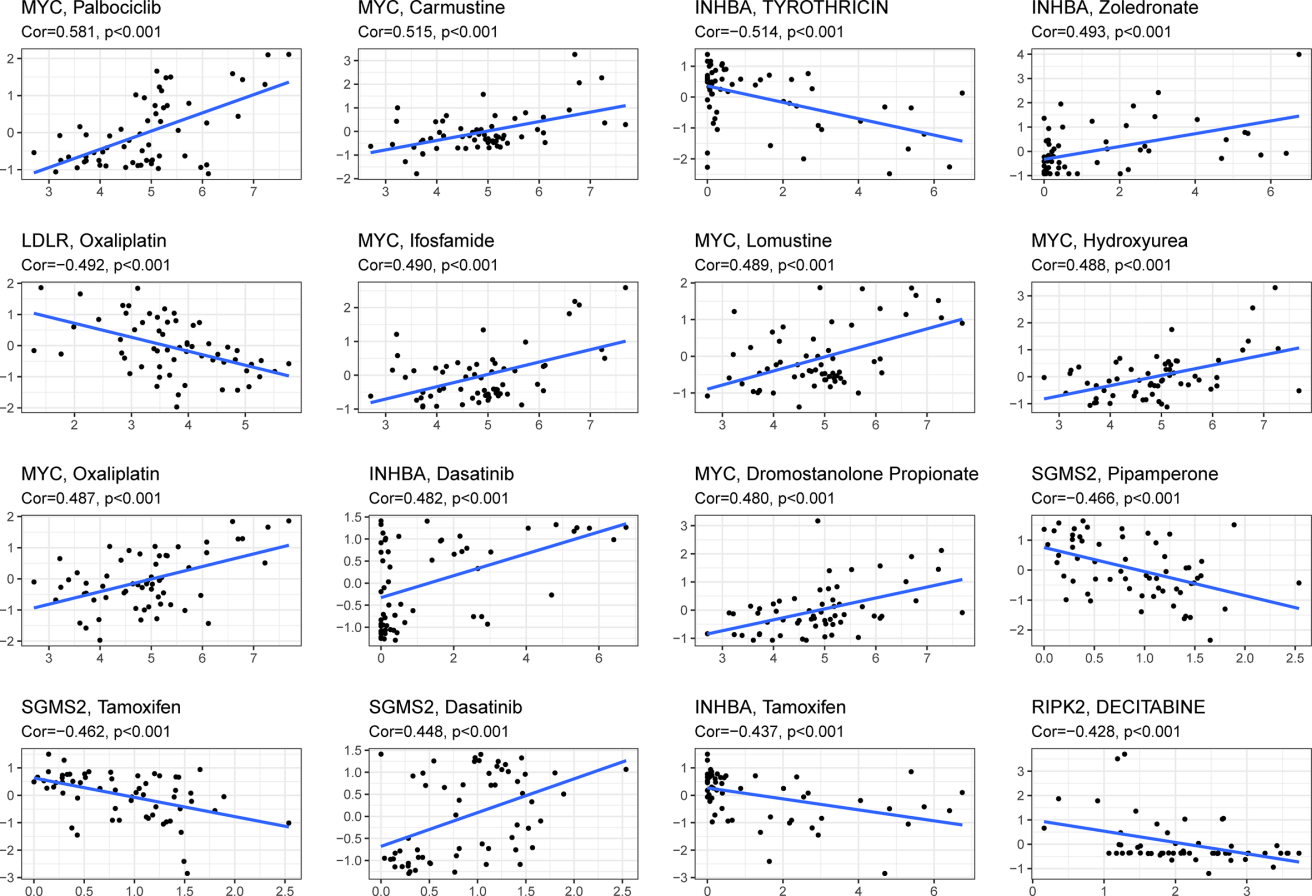
Scatter plot of relationship between prognostic gene expression and drug sensitivity.

To study the relationship between these prognostic genes and drug sensitivity, we determined their level in NCI-60, a public database of human cancer cell lines. The results showed the top 16 correlation analysis according to the p-value. **Figure 8** demonstrates that MYC is sensitive to Palbociclib, Carmustine, Ifosfamide, Lomustine, Hydroxyurea, Oxaliplatin, Dromostanolone Propionate (p < 0.001), and INHBA is sensitive to Zoledronate and Dasatinib (p < 0.001), while it is insensitive to Tyrothricin and Tamoxifen (p < 0.001). Besides, the expression of LDLR is insensitive to Oxaliplatin (p < 0.001), and RIPK2 is insensitive to Decitabine. Moreover, SGMS2 is sensitive to Dasatinib, while it is insensitive to Pipamperone and Tamoxifen (all p < 0.001).

### Verification of the Prognostic Gene Expression by qRT-PCR

**Figure 9:**
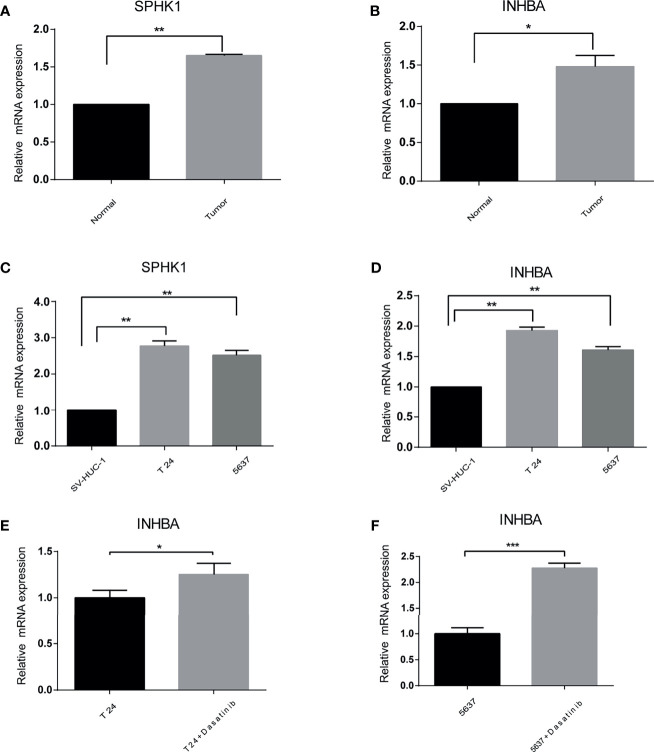
The difference of the prognostic gene expression was confirmed by qRT-PCR. **(A, B)** *In vivo*. **(C, D)** *In vitro*. **(E, F)** Presence or absence with Dasatinib *in vitro*. *p < 0.05, **p < 0.01, ***p < 0.001.

To validate the different expressions of these prognostic genes, qRT-PCR was implemented to analyze the mRNA expression *in vivo* and *in vitro*. The results of qRT-PCR showed that the mRNA expression of INHBA and SPHK1 were significantly increased in cancer than in normal tissues and cells (**Figures 9A–D**). In addition, we observed that the mRNA expression of INHBA was significantly upregulated in T24 and 5637 cells when treated by Dasatinib (**Figures 9E, F**).

## Discussion

In the era of personalized precision medicine, high-throughput sequencing technology had been widely used for treating cancer including bladder cancer (16). Nevertheless, due to the lack of useful biomarkers, it is difficult for us to make early diagnosis and evaluate the efficacy of treatment on BUC patients (17). Recent studies suggested that some serum biomarkers, such as circulating tumor cells, vitamin D, and circulating tumor DNA, have good accuracy of BUC diagnosis and prognosis (18–20). Moreover, in addition to early diagnosis, inflammatory indexes can also contribute to predict prognosis of BUC (10, 21, 22). Nevertheless, the IRAGs has not yet been reported as a prognostic marker for BUC. Recent studies have demonstrated that ferroptosis, EMT, glycolysis, m6A, and hypoxia-associated gene signatures had been used for predicting OS of BUC, which were consistent with our study (23–26). In addition, comparing with risk gene signatures above, the IRAGs model constructed in our study had more advantages. For example, our study proved that the prognostic IRAGs were related to drug sensitivity and resistance.

Two hundred IRAGs were enrolled into the current study to determine the expression level of BUC patients and the association with prognosis. Forty-nine differentially expressed IRAGs were filtrated from the TCGA cohort. Twelve of these genes were related to the prognosis in BUC patients by univariate Cox regression analysis. We used TCGA cohort to develop a novel risk model based on 10 prognostic IRAGs and GSE 13507 cohort to validate the reliability. Based on the risk score, we split BUC patients in TCGA or GSE 13507 cohort into two groups. Patients with low-risk score are more likely to have a lower tumor grade, tumor stage, and longer survival time. Univariate and multivariate survival analysis demonstrated that the risk score was an independent prognostic indicator for BUC patients.

Ten IRAGs were included in the model (CXCL11, INHBA, LDLR, MMP14, MYC, PTGER4, RIPK2, SGMS2, SPHK1, and TNFAIP6). C-X-C motif chemokine 11 (CXCL11) has been reported to contribute to the progression of tumors. In our study, we demonstrated that the expression of CXCL11 was negatively correlated with prognosis of BUC, which was consistent with the result of Vollmer Tino et al. In addition, they also found that the expression of CXCL11 was predictive of chemotherapy response in human bladder cancer (27). We also demonstrated the correlation between CXCL11 and chemotherapy drugs in this study, which was consistent with previous studies. Inhibin subunit beta A (INHBA) is highly expressed in various tumors. In our study, we also observed that the expression of INHBA was significantly increased in BUC than in normal tissues or cells. Moreover, our study showed that the high expression of INHBA was significantly associated with poor prognosis of BUC, which was also demonstrated by Sugawara Sho et al. (28). In terms of drug sensitivity, we observed that the expression of INHBA was positively correlated with Dasatinib. Besides, we also observed the increased expression of INHBA after treatment with Dasatinib *in vitro*.

Low-density lipoprotein receptor (LDLR) is differentially expressed in bladder cancer. We observed that the expression of LDLR was negatively correlated with the sensitivity of Oxaliplatin. Moreover, Hamm et al. revealed cholesterol biosynthesis as an important resistance mechanism in T24 cells after archazolid B treatment (29). Our results demonstrated that the expression of MMP14 was associated with the prognosis in transitional bladder cancer. In addition, Wang et al. demonstrated the downregulation of MMP14-suppressed BC cell invasion and migration abilities *in vitro* (30). Moreover, our study demonstrated the expression of MMP14 was related to many chemotherapeutic drugs. A previous study has demonstrated that MMP was sensitive to the DNA demethylation molecule 5-aza-2′-deoxycytidine in T24 and 5637 cells (31). Therefore, MMP14 may be the therapeutic target for BUC patients in the future.

MYC is a proto-oncogene. Comparing with normal tissues, the expression of MYC in mRNA level was significantly increased, which may promote the development for BUC patients. Our results also demonstrated that the expression of MYC was related to the prognosis in BUC patients. In addition, we observed that the expression of MYC was positively associated with drug sensitivity. It has been reported that thymoquinone can inhibit the expression of MYC gene and then suppress invasion and metastasis in bladder cancer cells (32). Prostaglandin E receptor 4 (PTGER4) is one of the receptors of prostaglandin E2. Musser et al. demonstrated that the transitional cell carcinoma tissues displayed significantly less mRNA EP4R expression when compared to normal bladder mucosa, which was consistent with our result (33). Besides, we demonstrated that PTGER4 is a protector in BUC patients. However, previous studies have illustrated that the expression of PPTGER4 was associated with the development of malignancy and a poor prognosis in multiple human cancers (pathological function of prostaglandin E2 receptors in transitional cell carcinoma of the upper urinary tract). Therefore, the role of PTGER4 in bladder cancer needs further investigation.

Receptor-interacting serine/threonine-protein kinase 2 (RIPK2) was markedly increased in bladder cancer and a protector as demonstrated by univariate Cox analysis. However, RIPK2 polymorphism was also involved in the development of bladder cancer (34). Sphingomyelin synthase 2 (SGMS2) is a transferase that regulates the synthesis of sphingomyelin from ceramide. In our study, we observed the decreased expression of SGMS2 in BUC. Zheng et al. have demonstrated that the high expression of SGMS2 was associated with breast cancer metastasis (35). In addition, we observed that SGMS2 was a risk factor by univariate Cox analysis, and the expression of SGSM2 was negatively correlated with drug sensitivity to Tamoxifen. Sphingosine kinase 1 (SPHK1) can phosphorylate sphingosine to form sphingosine-1-phosphate, which plays critical roles in the regulation of cancer cell proliferation and survival in different types of cancer (36). Our study demonstrated that the increased expression of SPHK1 and SPHK1 was a prognostic biomarker in bladder cancer. miR163 and miRNA 125b can inhibit bladder cancer proliferation and migration through targeting SPHK1. Our results demonstrated that tumor necrosis factor (TNF) alpha-induced protein 6 (TNFAIP6) was one of the differentially expressed genes. It has been reported that TNFAIP6 high-expression predicted poor OS in patients with urothelial carcinoma (37). Likewise, we demonstrated that TNFAIP6 was a risk factor by survival analysis in our study.

To have a better understanding of the role of risk score in immune infiltration, six were involved in this study. The results showed that patients in high-risk group may happen with the enrichment of C1 in their microenvironment, while those with low-risk score usually happened with C2, C3, and C4 enrichment, which means that C1 is a risk factor and the other types can inhibit the genesis and progression of transitional bladder cancer. Since a high cytotoxicity can suppress the genesis and progression of tumor, the results of our study were in accordance with these previous findings (38). In relation to clinical implication, patients in high-risk groups are more likely to have higher tumor stage, which was a risk factor for cancer.

According to the results of GSEA, mitogen-activated protein kinase (MAPK), and Wnt signaling pathways were markedly enriched, which had been proven to be correlated with cancer, and they may be the potential therapeutic target in the future. Moreover, signal pathways such as melanoma, pathways in cancer, basal cell carcinoma, and colorectal cancer were enriched in high-risk groups, which demonstrated the important role of these IRAGs in cancer. Furthermore, patients are more likely to have a higher score of mast cells and macrophages, while they had lower scores of NK cells than the low-risk group. Interestingly, previous studies suggested that NK cells are a potent class of antitumor cell in bladder cancer, which was similar to our results (39). Besides, the risk score was markedly associated with stromal score, which means that tumor environment may contribute to the aggression of bladder cancer and negatively relate with RNAss that may be a protector for bladder cancer.

By analyzing the data of 60 different cell lines, the elevated expression of these prognostic genes cannot only enhance the drug sensitivity but also increase the resistance of chemotherapy drugs approved by the Food and Drug Administration (FDA). For example, cancer cells were sensitive to Tyrothricin with the increased expression of INHBA genes, while they were insensitive to Zoledronate. Moreover, cells with increased expression of MYC genes were susceptible to Oxaliplatin, which had been approved by FDA for bladder cancer. Therefore, these data may provide a new sight for precision therapy in the future.

To further verify the reliability of our results, qRT-PCR was performed *in vivo* and *in vitro* for INHBA and SPHK1 genes. The expression levels of INHBA and SPHk1 were all markedly upregulated for bladder cancer both *in vivo* and *in vitro*. In addition, when treated by Dasatinib, the expression of INHBA was significantly increased in T24 and 5637 cells, which was consistent with the correlation between INHBA and Dasatinib.

A good predictive ability of the risk model and differential expression of prognostic genes have been demonstrated in transitional bladder cancer. Nevertheless, there are two limitations in our study. First, to achieve a comprehensive understanding of these prognostic IRAGs, we should conduct multi-omics analysis. Second, a large-sample multicenter study is required to demonstrate the findings of our study.

## Conclusions

Our prognostic model can accurately discriminate the transitional bladder cancer patients. Patients that have a worse survival time in high-risk groups can be identified by this model before the disease progression and obtained appropriate therapies immediately. In addition, cancer cells with these prognostic genes are sensitive or insensitive to FDA-approved antitumor drugs, which may contribute to the targeted therapies in transitional bladder cancer in the future.

## Data Availability Statement

The original contributions presented in the study are included in the article/**Supplementary Material**. Further inquiries can be directed to the corresponding authors.

## Ethics Statement

The studies involving human participants were reviewed and approved by the Ethics Committee of Shanghai General Hospital. The patients/participants provided their written informed consent to participate in this study.

## Author Contributions

JJ and AL conducted research and conceived the design. ZX and JC performed data collection and article writing. SH performed data analysis. WS revised the manuscript. All authors contributed to the article and approved the submitted version.

## Funding

This work was supported by The National Natural Science Foundation of China (No. 81771564) and Zunyi Municipal Science and technology Bureau [No. 2018(192)].

## Conflict of Interest

The authors declare that the research was conducted in the absence of any commercial or financial relationships that could be construed as a potential conflict of interest.

## Publisher’s Note

All claims expressed in this article are solely those of the authors and do not necessarily represent those of their affiliated organizations, or those of the publisher, the editors and the reviewers. Any product that may be evaluated in this article, or claim that may be made by its manufacturer, is not guaranteed or endorsed by the publisher.

## Supplementary Material

Supplementary Figure 1 Forest plots showing the results of the association between IRAGs and OS.

Supplementary Figure 2 AUC time-dependent ROC curves for OS in GSE13507 cohort.

Supplementary Table 1 The 200 inflammatory response-related genes.

Supplementary Table 2 Differentially expressed genes between HCC tissues and adjacent non-tumorous tissues in TCGA.

Supplementary Table 3 The primer sequences of the prognostic genes.

Supplementary Table 4 The level of prognostic genes in NCI 60 human cancer cell lines.

Supplementary Table 5 Marker genes for immune cell types.

## Abbreviations

IRAGs, inflammatory response-associated genes; MSigDB, Molecular Signature Database; TCGA, The Cancer Genome Atlas; BLCA, bladder urothelial carcinoma; LASSO, least absolute shrinkage and selection operator; OS, overall survival; qRT-PCR, quantitative reverse transcription polymerase chain reaction; ROC, receiver operating characteristic; BUC bladder urothelial carcinoma; GEO, Gene Expression Omnibus; ssGSEA, single sample Gene Set Enrichment Analysis; ANOVA, analysis of variance; RNAss, RNA stemness score; DNAss, DNA stemness score.
