# Supplementary figures and images for: Development and Validation of Prognostic Model in Transitional Bladder Cancer Based on Inflammatory Response-Associated Genes
Source: Front Oncol. 2021 Oct 7;11:740985. doi: 10.3389/fonc.2021.740985 (PMC8529162; doi:10.3389/fonc.2021.740985)

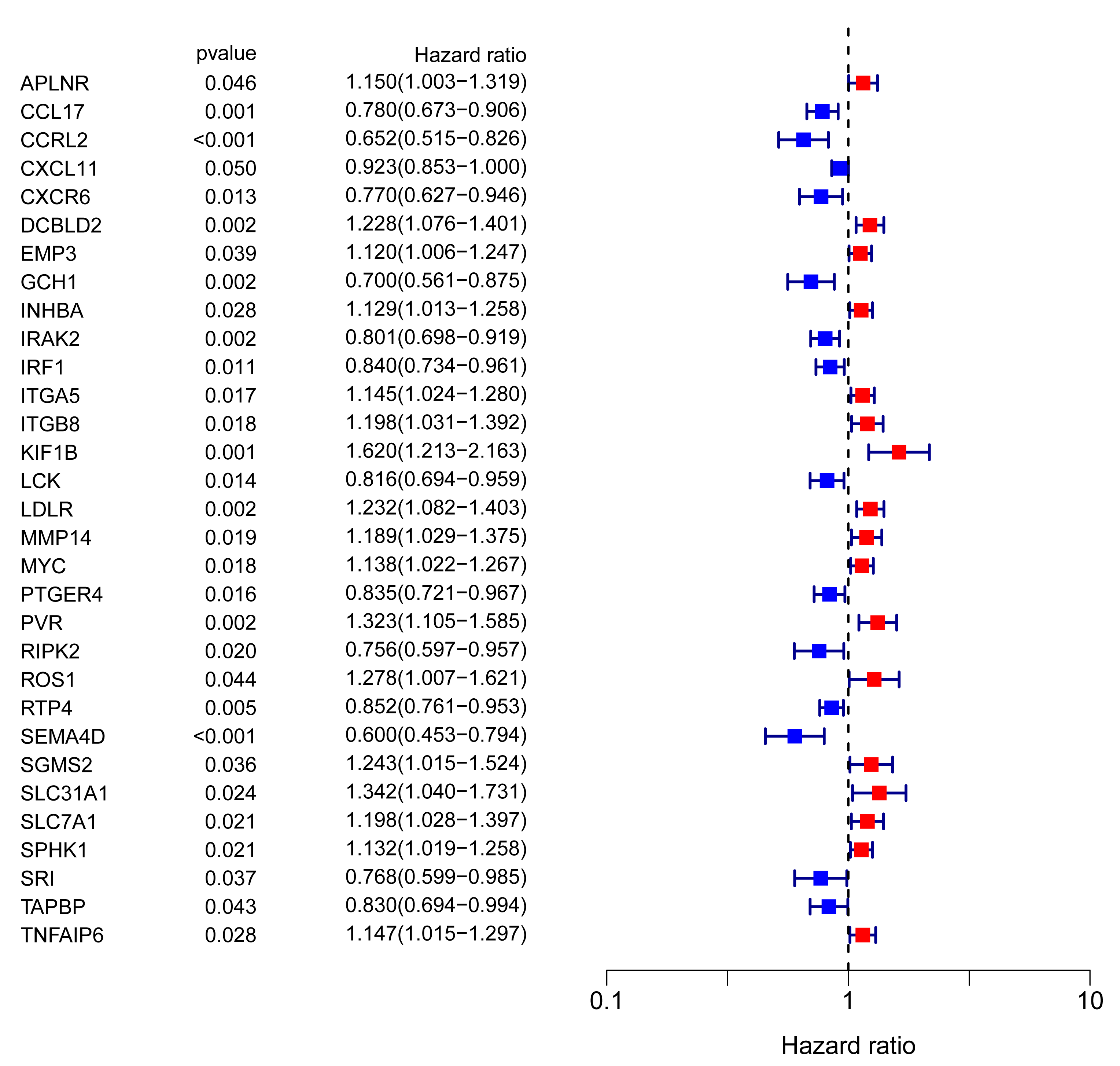

Supplement: Supplementary Figure 1 — Forest plots showing the results of the association between IRAGs and OS. [file Image_1.tif]

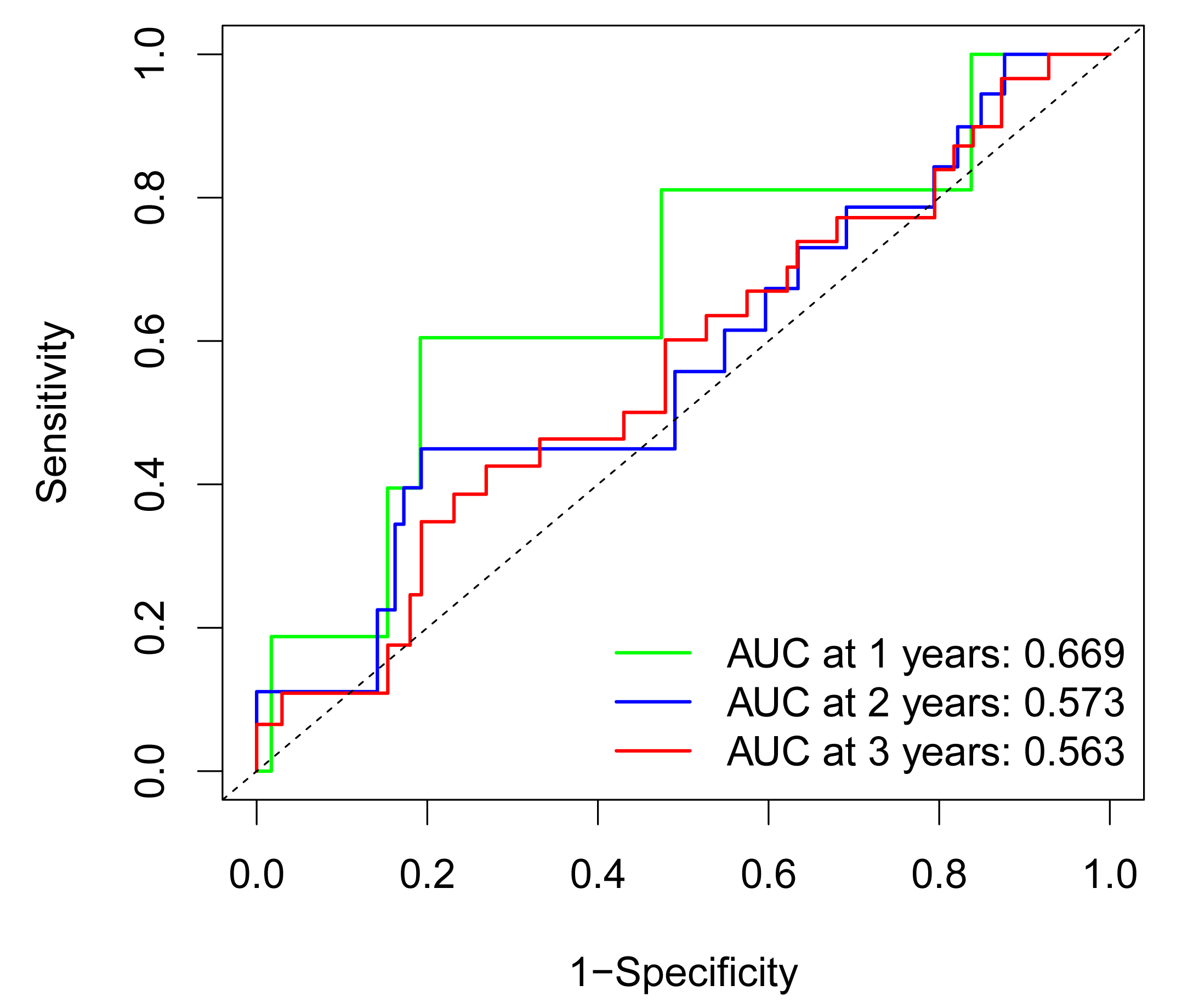

Supplement: Supplementary Figure 2 — AUC time-dependent ROC curves for OS in GSE13507 cohort. [file Image_2.tif]
