# Supplementary material for: Development and Validation of Prognostic Model in Transitional Bladder Cancer Based on Inflammatory Response-Associated Genes
Source: Front Oncol. 2021 Oct 7;11:740985. doi: 10.3389/fonc.2021.740985 (PMC8529162; doi:10.3389/fonc.2021.740985)
Supplement: Supplementary Table 3 — The primer sequences of the prognostic genes. [file Table_3.docx]

Table S3 The primer sequences of INHBA, SPHK1 and GAPDH

| Gene | Primer | Sequence (5’-3’) |
| --- | --- | --- |
| GAPDH | F primer | GTCTCCTCTGACTTCAACAGCG |
|  | R primer | ACCACCCTGTTGCTGTAGCCAA |
| INHBA | F primer | GGATGACATTGGAAGGAGGGCA |
|  | R primer | ACTGACAGGTCACTGCCTTCCT |
| SPHK1 | F primer | TGGTGAACGGGCTCATGGAG |
|  | R primer | AGGTCTTCATTGGTGACCTGCTC |

F primer, forward primer; R primer, reverse primer.
